# The Effect of Colloidal Nanoparticles on Phase Separation of Block and Heteroarm Star Copolymers Confined between Polymer Brushes

**DOI:** 10.3390/ma17040804

**Published:** 2024-02-07

**Authors:** Minna Sun, Wenyu Chen, Lei Qin, Xu-Ming Xie

**Affiliations:** 1 Beijing Key Laboratory for Sensors, Beijing Information Science and Technology University, Beijing 100192, China; sunminna@bistu.edu.cn; 2 Beijing Key Laboratory for Optoelectronic Measurement Technology, Beijing Information Science and Technology University, Beijing 100192, China; 2021020230@bistu.edu.cn; 3 Key Laboratory of Advanced Materials (MOE), Department of Chemical Engineering, Tsinghua University, Beijing 100084, China

**Keywords:** architecture, dispersion, heteronym star copolymers, colloidal nanoparticles, self-consistent field theory

## Abstract

The effect of colloidal nanoparticles on the phase changes of the amphiphilic AB linear diblock, A_1_A_2_B, and A_2_B heteroarm star copolymers confined between two polymer brush substrates was investigated by using a real-space self-consistent field theory. By changing the concentrations of nanoparticles and polymer brushes, the phase structure of the amphiphilic AB copolymer transforms from lamellar to core-shell hexagonal phase to cylinder phase. The pattern of A_2_B heteroarm star copolymer changes from core-shell hexagonal phases to lamellar phases and the layer decreases when increasing the density of the polymer brushes. The results showed that the phase behavior of the system is strongly influenced by the polymer brush architecture and the colloidal nanoparticle numbers. The colloidal nanoparticles and the soft confined surface of polymer brushes make amphiphilic AB copolymers easier to form ordered structures. The dispersion of the nanoparticles was also investigated in detail. The soft surfaces of polymer brushes and the conformation of the block copolymers work together to force the nanoparticles to disperse evenly. It will give helpful guidance for making some new functional materials by nano etching technology, nano photoresist, and nanoprinting.

## 1. Introduction

By introducing a small amount of colloidal particles into polymer materials, it is possible to significantly alter their properties across various domains, including electrical, thermal, and mechanical properties [1]. The doping of nanoparticles has emerged as a vital tool in the development of novel functional materials, particularly in areas such as nanoetching technology, nano-photoresists, and nanoprinting [2,3,4]. In recent years, there has been significant progress in the field of block copolymers and nanoparticles, which have garnered much attention both in experimental and theoretical research [5]. Various methods, such as confinement [6,7,8,9,10,11,12,13], external fields [14,15,16,17], patterned surfaces [18,19,20,21], and surface topography [22,23,24,25], have been utilized to investigate their properties and behaviors. The colloidal particles dispersed in polymer solutions serve as fundamental components in numerous vital and essential industrial systems. Notably, the self-assembly of colloidal nanoparticles into ordered structures plays a pivotal role in the production of nanomaterials endowed with superior optical, electrical, and mechanical properties. In this field, a formidable challenge lies in achieving a highly ordered structure of monodisperse colloidal particles and precisely manipulating their size and shape [26]. Typically, the structure of colloidal nanoparticle dispersion is determined by the effective interactions between colloidal particles and the interaction between the colloid and its surrounding environment. The interaction of colloid nanoparticles in non-attractive polymer solutions is governed by the entropy of the polymer chains, which creates a depletion force. As a result, colloidal crystallization leads to the formation of a typical structure with high symmetry: the body-centered cubic structure and dense packing structure.

The combination of polymers and colloidal particles can produce fascinating structures, particularly in the field of nanotechnology, and nanopatterning/photolithography, by changing the surface properties. The surface constraint alters the phase behavior of the system by disrupting the symmetric structure, enabling the creation of low-dimensional materials. The grafting of polymer brushes onto surfaces can modify surface properties, leading to numerous applications in colloid stability and biocompatibility [27]. For instance, block copolymers confined between two solid surfaces can form a lamellar phase, either parallel or perpendicular to the substrate surfaces, depending on the wetting properties of the confining surfaces [28]. However, the interfacial environment of confining surfaces is a significant factor influencing phase behavior. Substrate surfaces grafted with polymers create an easily deformable “soft” wall instead of a rigid one, altering the surface environment [29,30,31]. When it comes to soft wall confinement, most studies have focused on the impact of polymer brush density. Researchers have observed a range of structures that depend on the compatibility of the film thickness [32,33,34]. In reality, the architecture of block copolymers plays a pivotal role in controlling phase behavior [35,36]. Matsen et al. pointed out that a good way to control the phase behavior of the block copolymers is by changing the properties of the surface of the parallel plates [25]. Polymer brushes grafted in the interface are an effective way to change the interface properties. Polymer brushes can change surface properties such as viscosity, lubrication, infiltration, and so on. These properties are mainly related to the shape of the polymer, such as the grafting density, the brush height, and the distortion behavior of the polymer brushes [37,38,39,40]. There are numerous practical applications for modifying surface properties, including colloidal stabilization, polymerized surface modifiers, and numerous other techniques. Ren [22] studied the phase behavior of the colloidal/polymer mixing between the polymer brush plates. They found that the system produced phase behavior transition from disordered liquid to sparse square to hexagonal (or square and hexagonal mix) to dense square to tubular with increasing colloidal concentration [41,42]. The aforementioned studies primarily concentrate on two-block copolymer systems, it is important to pay attention to the dispersion of colloidal nanoparticles in systems with well-distributed and micelle properties of the three-block copolymers [42,43,44,45]. The interaction between the colloidal nanoparticle dispersion and the copolymer architecture holds great significance. In this study, we employed self-consistent field methods to delve into the phase morphology of amphiphilic copolymers, specifically the linear diblock A_1_A_2_B and the heteroarm star copolymer A_2_B [46,47,48,49]. We also considered the impact of colloidal nanoparticle dispersion in systems confined by grafted homopolymer chains. The three types of block copolymers can be conceptualized as distinct variations in the movement of the branch point along the chain [50]. Our findings reveal that, for a fixed volume fraction and grafting density, the system undergoes a phase transition from layers to columns as the position of the linked branch point is gradually shifted [51]. 

## 2. Model and Method

The effect of colloidal nanoparticles on phase separation of AB linear block copolymer and A_1_A_2_B/A_2_B heteronym star copolymers confined between polymer brushes was investigated by the self-consistent field theory. The system of *n_co_* copolymers is confined between two planar surfaces with a distance *L_z_* along the *z*-axis [37,38,39,40]. The copolymers have the same polymerization N and different architecture. The substrate surface is grafted with *n_br_* homopolymer(denoted by *H*) chains of polymerization *N_H_* (*N_H_* = *N*). The homopolymer brushes (*H*) are horizontally placed in the *x-y* plane and positioned at *Z* = 0 and *Z* = *L_z_*, respectively. It assumes translation invariance along the y-axis. The fractions of A and B segments are f_a_ and f_b_, respectively. If the architecture of the copolymer is heteroarm star, the A block is divided into two segments A_1_ and A_2_, then the fraction of the A segment is *f_a_ = f_a1_ + f_a2_* [41,42,43]. All polymer chains are flexible with the same statistical length a, and incompressible with a segment volume *ρ_0_^−1^*. N=100, the constant scale of the polymer is $\bar{N}=a^{6}\rho_{0}^{2}N$. Where a is the Kuhn length of the polymer segment. The radius of the nanoparticle (denoted by P) is *r_p_*, and the volume of the nanoparticle is $\nu_{r}=4\pi r_{p}^{3}∕3$. The volume of the system *V* is L_x_ × *L_z_*, where *L_x_* and *L_z_* are the lengths of the surfaces along the *x*-axis and the *z*-axis, respectively. The grafting density is *σ = n_h_/L_x_*, and *n_h_* denotes the number of the homopolymer brushes. The volume fraction of the grafted chain is $\phi_{h}$. The volume fraction of the total volume occupied by the particles is $\;\phi_{p}$. The average volume fractions of the grafted chains and block copolymers are $\bar{\phi_{h}}=n_{co}N\rho_{0}/V$ and $\bar{\phi_{co}}=n_{co}N\rho_{0}^{-1}/V$, respectively. $\chi_{ij\;}$ is the Flory-Huggins interaction parameter of the system, where *i* and *j* represent A, B, H, and P, respectively. Which $\chi_{ab\;}=\chi_{bp\;}=\chi_{bh\;}=20$, $\chi_{ah\;}=\chi_{ap}=\chi_{hp\;}=0$.

The volume ratio of the colloidal nanoparticles and the block copolymers is α, then

(1) $$\alpha \equiv \frac{v_{r}\rho_{0}}{N}=\frac{4\pi }{3}(\frac{r_{p}}{R_{0}}{)}^{3}{\bar{N}}^{\frac{1}{2}}$$

where $R_{0}=\alpha N^{\frac{1}{2}}\;$ represents the mean square end distance of the polymer, the system free energy is obtained by the mean-field approximation as follows:

(2) $$\begin{array}{cc} \; & \frac{NF}{\rho_{0}k_{B}TV}=-\phi_{co}\ln\left(\frac{Q_{co}}{V\phi_{co}}\right)-\phi_{h}\ln\left(\frac{Q_{h}}{V\phi_{h}}\right)-\frac{\phi_{p}}{\alpha }\ln\left(\frac{Q_{p}\alpha }{V\phi_{p}}\right)+\frac{1}{V}\int dr[\chi_{ab}N\varphi_{a}\left(r\right)\varphi_{b}\left(r\right)+\\ \; & \chi_{bh}N\varphi_{b}\left(r\right)\varphi_{h}\left(r\right)+\chi_{bp}N\varphi_{b}\left(r\right)\varphi_{p}\left(r\right)-w_{a}\left(r\right)\varphi_{a}\left(r\right)-w_{b}\left(r\right)\varphi_{b}\left(r\right)-w_{h}\left(r\right)\varphi_{h}\left(r\right)\\ \; & -w_{p}\left(r\right)\rho_{p}\left(r\right)-\xi \left(r\right)\left(1-\varphi_{a}\left(r\right)-\varphi_{b}\left(r\right)-\varphi_{h}\left(r\right)-\varphi_{p}\left(r\right)\right)+\rho_{p}\psi_{hs}\left(\bar{\varphi_{p}}\right)] \end{array}$$

where $Q_{co}$, $Q_{h}$ and $Q_{p}$ are the single chain partition functions of grafted homopolymers brushes, the block copolymers, and nanoparticles, respectively. $\varphi_{a}(r)$, $\varphi_{b}(r)$, $\varphi_{h}\left(r\right),$ and $\varphi_{p}(r)$ are the local volume fraction of the grafted homopolymers, A segments, B segments, and nanoparticles, respectively. The $w_{a}(r)$, $w_{b}\left(r\right)$, $w_{h}(r)$ and $w_{p}(r)$ are the potential fields of A, and B segments of the block copolymers, the grafted homopolymers and the nanoparticles, respectively. 

In this work, the nanoparticles are hard balls. The steric free energy of the nanoparticle is $\psi_{hs}(\bar{\varphi_{P}})$*,* which can be calculated by the Carnahan-Starling function.

(3) $$\psi_{hs}(x)=\frac{4x-3x^{2}}{(1-x{)}^{2}}$$

The localized volume fraction and weight non-localized volume fraction are presented as follows:

(4) $$\phi_{p}(r)=\frac{\alpha }{v_{r}}\int_{\left|r^{’}\right|<r_{p}}dr^{’}\rho_{p}(r+r^{’})$$

(5) $${\bar{\phi }}_{p}(r)=\frac{\alpha }{v_{2r}}\int_{\left|r^{’}\right|<2r_{p}}dr^{’}\rho_{p}(r+r^{’})$$

In the above equation, $\rho_{P\left(\gamma \right)}$ is the nanoparticle center distribution. $v_{2r}$ is the space volume of a nanoparticle sphere. By minimizing the free energy of Equation (2) with the monomer densities and mean fields, it leads to the set of mean-field equations:

(6) $$w_{a}(r)=\chi_{ab}N\varphi_{b}+\xi (r)$$

(7) $$w_{h}(r)=\chi_{bh}N\varphi_{b}+\xi (r)$$

(8) $$w_{b}(r)=\chi_{ab}N\varphi_{a}+\chi_{bh}N\varphi_{h}+\chi_{bp}N\varphi_{p}+\xi (r)$$

(9) $$w_{p}(r)=\psi_{hs}(\bar{\varphi_{p}}(r))+\frac{\alpha }{v_{r}}\int_{\left|r^{’}\right|<r_{p}}dr^{’}[\chi_{ap}N\varphi_{a}(r+r^{’})+\chi_{bp}N\varphi_{b}(r+r^{’})+\chi_{hp}N\varphi_{h}(r+r^{’})+\xi (r+r^{’})]+\frac{\alpha }{v_{2r}}\int_{\left|r^{’}\right|<2r_{p}}dr^{’}[\rho_{p}(r+r^{’})\psi_{hs}^{’}((\bar{\varphi_{p}}(r+r^{’}))]$$

(10) $$\phi_{a}(r)=\frac{(1-\varphi_{p}-\varphi_{h})V}{Q_{co}}[\int_{0}^{f_{a1}}dsq_{a1}(r,s)q_{a1}^{+}(r,s)+\int_{0}^{f_{a2}}dsq_{a2}(r,s)q_{a2}^{+}(r,s)]$$

(11) $$\phi_{b}(r)=\frac{(1-\varphi_{p}-\varphi_{h})V}{Q_{co}}\int_{0}^{f_{b}}dsq_{b}(r,s)q_{b}^{+}(r,s)$$

(12) $$\phi_{h}(r)=\frac{\varphi_{h}V}{Q_{h}}\int_{0}^{1}dsq_{h}(r,s)q_{h}^{+}(r,s)$$

(13) $$\rho_{p}(r)=\frac{\varphi_{p}V}{\alpha Q_{p}}\exp[-w_{p}(r)]$$

(14) $$\varphi_{a}(r)+\varphi_{b}(r)+\varphi_{h}(r)+\varphi_{p}(r)=1$$

(15) $$Q_{co}=\int drq_{k}(r,s)q_{k}^{+}(r,s)\left(k=a_{1}/a_{2}/b\right)$$

(16) $$Q_{h}=\int drq_{h}(r,s)q_{h}^{+}(r,s)$$

(17) $$Q_{p}=\int dr\exp\left[-w_{p}(r)\right]$$

*ξ(r)* is the Lagrange multiplier to ensure the incompressibility of the system [39,47,48]. 

$q_{i}(r,s)$ and $q_{i}^{+}(r,s)$ are the end-segment distribution functions that represent the probability of finding the *s* segment at position *r* from two distinct ends of chains, they satisfied the modified diffusion equations:

(18) $$\frac{\partial q^{+}(r,s)}{\partial s}=-(\frac{a^{2}N}{6})\nabla^{2}q^{+}(r,s)+w(r)q^{+}(r,s)$$

(19) $$\frac{\partial q(r,s)}{\partial s}=(\frac{a^{2}N}{6})\nabla^{2}q(r,s)-w(r)q(r,s)$$

The grafted chains in the field $w_{h}(r)$, the initial condition is: $q_{h}(r=r_{z},0)=1$, $q_{h}(r\neq r_{z},0)=0$, and $q_{h}^{+}(r,1)=1$*,* here, *r_z_* presents the positions of two substrates at *z = 0* and *z = Lz*. 

The polymer chains are limited in the region *0 ≤ z ≤ L_z_*. The modified diffusion Equations (18) and (19) were solved by the Crank-Nicholson scheme and the alternating-direct implicit (ADI) method. Minimizing the free energy in Equation (2) with respect to *φ_a_(r), φ_b_(r), φ_h_(r),* $w_{a}(r)$*,* $w_{b}(r)$*,*$\;w_{h}(r)$ and $w_{p}(r)$, the free energy could be obtained.

The radius of the particle is $0.3R_{0}$, the self-consistent Equations (6)–(17) were solved by the real space combinatorial screening algorithm of Drolet and Fredrickson [33,34]. The volume fractions were obtained from Equations (10)–(12), and the fields were calculated by Equations (6)–(9). Then updated new fields were involved in Equations (15)–(17). Then the calculation was simulated by the Fortran program. Periodic boundary conditions were performed along the x-axis. In the program, an initial value of the concentration distribution of block and heteroarm star copolymers in the system was set at random due to the phase separation affected by fluctuations [49]. The calculation was started using initial random values of the field. The equilibrium convergence condition of the system is the difference of the free energy ΔF  less than $\;10^{-5}$. For the convenience and simplicity of calculation, it is assumed that the concentration distribution in the y direction is constant. Therefore, only the concentration distribution and field changes in the *x-z* plane need to be calculated.

## 3. Numerical Results and Discussions

**Figure 1:**
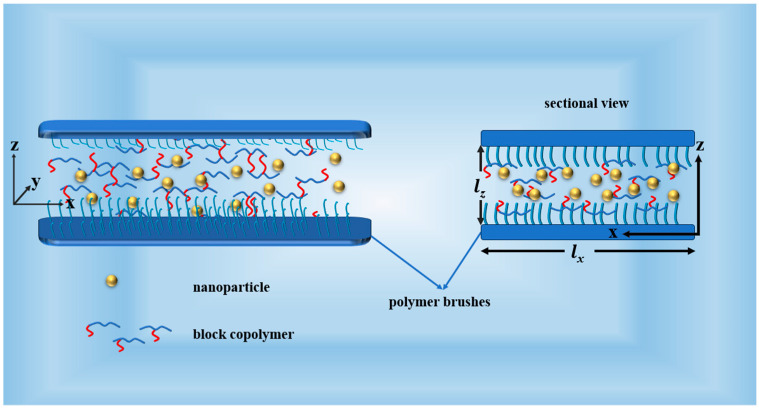
Schematic diagram of the AB dilock copolymers, A_1_A_2_B and A_2_B heteroarm star copolymers, and colloidal nanoparticles mixture confined in a substrate with polymer-grafted brushes. In the A segment of the copolymer and the B segment of the copolymer, the nanoparticles and brushes are represented in blue, red, gold, and little blue, respectively.

Figure 1 shows the schematic diagram of the system of the AB dilock copolymers, A_1_A_2_B and A_2_B heteroarm star copolymers, and colloidal nanoparticles mixture confined between two homopolymer-grafted substrates. In the A segment of the copolymer and the B segment of the copolymer, the nanoparticles and brushes are represented in blue, red, gold, and little blue, respectively. The parameters were set as $\bar{\phi_{h}}$ = 0.6, $f_{a}\;$= 0.6, $\chi_{ab}N$ = 20, $L_{x}=L_{z}$ = 100, N = 100. Here, the volume fraction of A segment is f_a_ = 0.6, A_1_A_2_B denotes the architecture of the block copolymers, *f_a_ = f_a1_ + f_a2_*, *f_b_ = 0.4.* With increasing f_a1_ from 0 to 0.3, it means that the B segment moves from the end of the A segment to the middle of the A segment. The architecture of AB copolymers also changed from AB diblock copolymers to A_1_A_2_B grafted copolymers to A_2_B star copolymers. The architecture of copolymers was investigated by changing the f_a1_.

**Figure 2:**
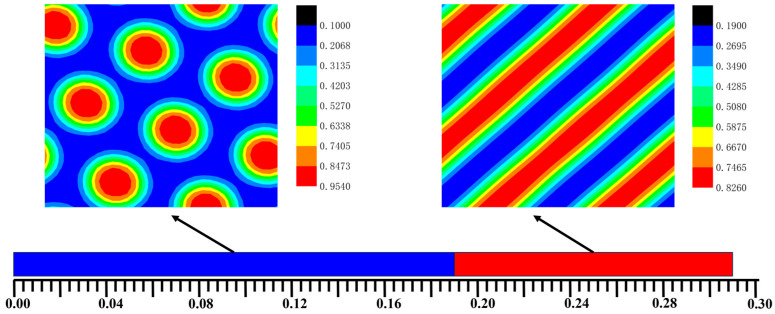
The bulk phase diagram of the system of the AB dilock copolymers, A_1_A_2_B and A_2_B heteroarm star copolymers. The hexagonal phases were formed when *f_a1_* < 0.2. The lamellar phases were formed when *f_a1_* > 0.2. The blue and red areas denote the A and B, respectively. The green area represented the interface of the bulk of A and B.

Figure 2 shows the bulk phase diagram of the system of the AB, A_1_A_2_B, and A_2_B heteroarm star copolymers. The hexagonal phases were formed when *f_a1_ < 0.2*. The lamellar phases were formed when *f_a1_ ≥ 0.2*. The blue and red areas denote the A and B, respectively. The green area represented the interface of the bulk of A and B. The bulk of the AB copolymers form a hexagonal phase generally that *f_a1_ =* 0. The bulk of the A_1_A_2_B copolymers forms a hexagonal phase that *f_a1_ =* 0.01~0.19. The bulk of the A_2_B copolymers forms a lamellar phase that *f_a1_ =* 0.2~0.3. Symmetric diblock copolymers are prone to form lamellar phases in the bulk, while asymmetric diblock copolymers tend to form columnar phases. The A_2_B star-block copolymers, which are highly symmetric, are likely to form lamellar phases.

### 3.1. The Phase Separation of the AB Diblock Copolymers, A_1_A_2_B, and A_2_B Heteroarm Star Copolymers

In this section, the fraction of the A segment, the concentration of the A segment, the B segment, and the polymer brushes H were systematically considered to investigate the effect of the architecture of AB, A_1_A_2_B, and A_2_B copolymers confined in a soft inner surface with polymer-grafted brushes. 

**Figure 3:**
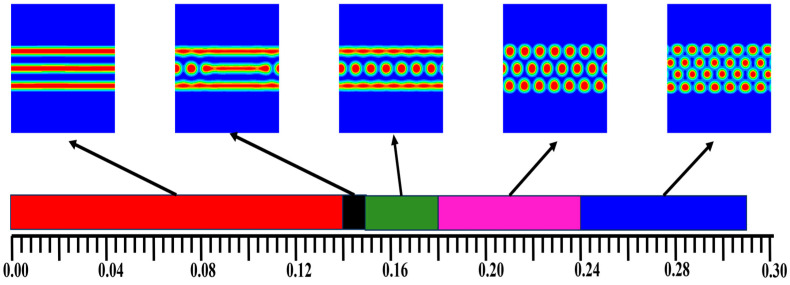
The phase diagram of the morphology of the AB diblock copolymers, A_1_A_2_B, and A_2_B heteroarm star copolymers with architecture changes by varying the *f_a1_*. The blue and red areas denote A and B, respectively.

Figure 3 shows the phase diagram of the morphology of the AB, A_1_A_2_B, and A_2_B block copolymers with architecture changes by varying the *f_a1_*. With the different grafting points, the system experienced a process from layered structure to layered and cylinder structure to three-cylinder structure and finally to four-cylinder structure.

**Figure 4:**
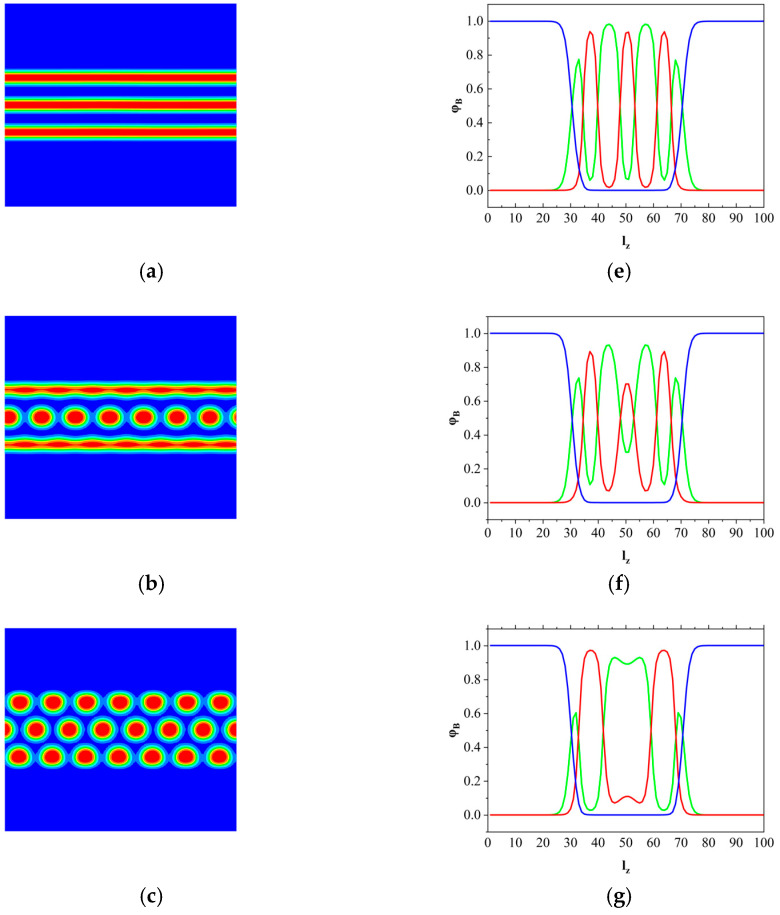

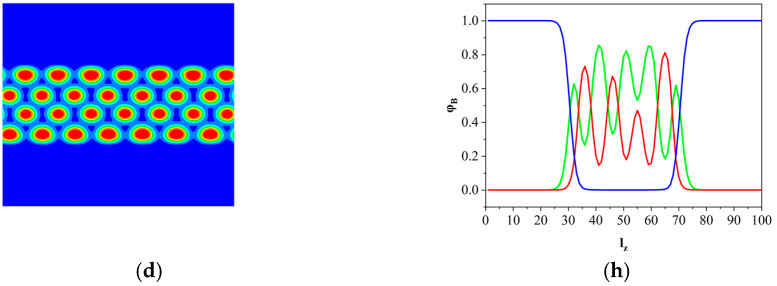
The morphology of the B phase at the x-z plane with different f_a1_. (**a**) $f_{a1}$ = 0.0, (**b**) $f_{a1}$ = 0.15, (**c**) $f_{a1}$ = 0.2, (**d**) $f_{a1}$ = 0.3. The corresponding x-direction-averaged profiles of $\varphi_{h}$*,* $\varphi_{a}$, and $\varphi_{b}$ along the z-axis. (**e**–**h**) The blue, green, and red lines denote the $\varphi_{h}$*,* $\varphi_{a}$*,* and $\varphi_{b}$, respectively.

Figure 4 shows the morphology of block copolymers changes from lamellar to hexagonal with different architectures of AB, A_1_A_2_B, and A_2_B copolymers. The snapshots of the left-hand side show the lamellar-hexagonal phase transition by increasing the fraction of *f_a1_*. On the right-hand side of Figure 4, the corresponding density profiles for the polymer-grafted brushes (*φ_h_*), A blocks (*φ_a_*), and B blocks (*φ_b_*) are given along the *z*-axis. In Figure 4a, the AB linear diblock copolymers are confined between polymer-grafted surfaces, and the block copolymers can be seen as near-symmetrical due to the fraction of A block is 0.6. The system forms a three-layered lamellar phase parallel to the surface, whereas the polymer brushes are strongly stretched and prefer to form a flat interface. In Figure 4b, by increasing the *f_a1_* to 0.15, the morphology of the system forms the lamellar with cylinder structures due to the effects of the architecture of the copolymer and the stretch of polymer brushes. In this case, the A_1_A_2_B architecture copolymer is prone to cylinder structures, whereas the polymer brushes tend to stretch along the *z* direction and the brushes interface remains flat, as shown in Figure 2. Thus, the A domains closing to brush interfaces tend to form lamellar, whereas the cylinder phase is formed at the middle region due to the architecture of the copolymer. With increasing the fraction of A_1_ block, the architecture of block copolymers is dominant in phase separation, which tends to form the three-layered cylinder structure [Figure 4c]. In this case, the brushes-formed interface will be deformed to maintain the system’s hexagonal phases as shown in Figure 4d. If it continues to increase *f_a1_*, the system forms a four-layered cylinder phase.

**Figure 5:**
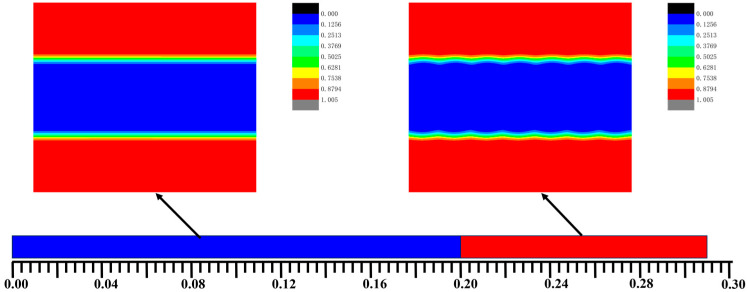
The morphology of the polymer brushes with different architectures of AB, A_1_A_2_B, and A_2_B copolymers. The interface of the polymer brush phases and the copolymers is smooth when *f_a1_* < 0.2. The interface of the polymer brush phases and the copolymers is rough edges when *f_a1_* > 0.2. The blue and red areas denote the copolymers and the polymer brushes, respectively.

Figure 5 shows the morphology of the polymer brushes with different architectures of copolymers. The interface of the polymer brush phases and the copolymers is smooth when *f_a_*_1_ *<* 0.2. The interface of the polymer brush phases and the copolymers is rough edges when *f_a_*_1_ *>* 0.2.

To clarify the interfacial energetic effects of polymer brushes on the phase behavior of block copolymer, the interfacial energy *F_int_* of polymer-grafted surfaces was calculated. The interfacial energy comes from the unfavorable contacts between grafted chains and the B component of copolymers. It is given by

(20) $$\frac{F_{int}}{K_{B}T}=\frac{\rho_{0}}{N}\int dr\chi_{ab}N\varphi_{h}\varphi_{b}$$

**Figure 6:**
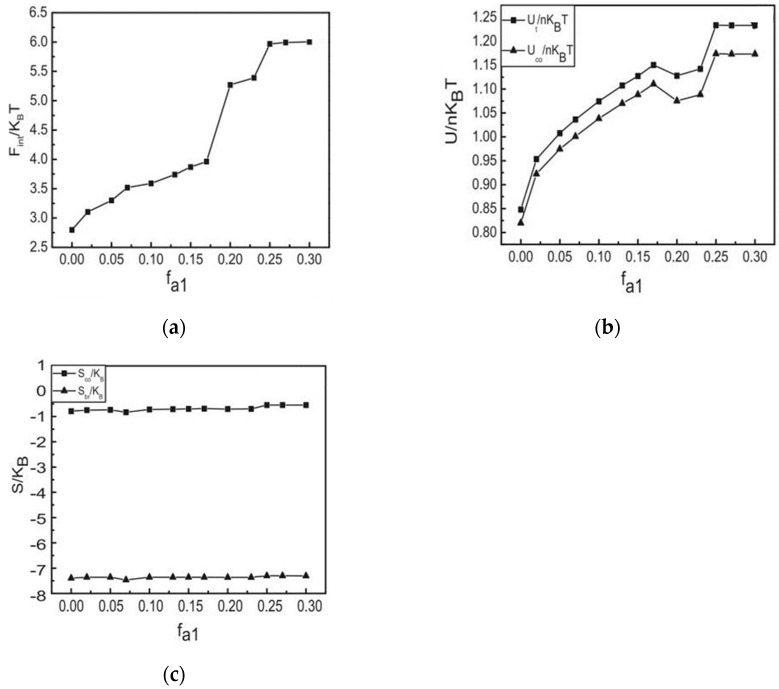
(**a**) The interfacial energy of the polymer brushes-formed surfaces as a function of the different architecture copolymers. (**b**) The internal energy of the system and the copolymer as a function of the different architecture copolymers. (**c**) The entropy of the block copolymer and the entropy of the polymer brushes as a function of the different architecture copolymers.

Figure 6a shows the interfacial energy of polymer brushes formed surfaces as a function of the fraction of block A_1_. From Figure 6a, there are two abrupt increases occur with the morphology changing from Figure 4b–d. By increasing the fraction of block A_1_, the brushes formed surface energy goes up. It means that the area of contact between the B phase and the polymer brushes increases. Thus, the protection of the B chain by the A chain is less efficient when the grafted point moves to the midpoint of the A chain. This is consistent with the domain morphology changing. The interface energy of the star copolymers is greater than the straight chain of copolymers.

It can be helpful to calculate the internal of the system (*U_t_*) and the internal of the block copolymer (*U_co_*), they can be expressed as:

(21) $$\frac{U_{t}}{nK_{B}T}=\frac{1}{V}\int dr[\chi_{ab}(\varphi_{a}(r)+\varphi_{h}(r))\varphi_{b}(r)]$$

(22) $$\frac{U_{co}}{nK_{B}T}=\frac{1}{V}\int dr[\chi_{ab}\varphi_{a}(r)\varphi_{b}(r)]$$

Figure 6b presents the internal energy of the system and the copolymer as a function of various architectures such as AB, A_1_A_2_B, and A_2_B copolymers. As observed in Figure 6b, the internal energy of the system (*U_t_*) and the copolymer (*U_co_*) exhibit a similar trend, indicating that the internal energy of the system is primarily contributed by the block copolymer. The internal energy of the copolymers plays a crucial role in determining the final structures. This is because the internal energy of the system is partitioned into the internal energy of the block copolymers and the internal energy of the grafted homopolymer brushes. The concentration of the polymer brushes remains unchanged, so the internal energy of the brushes does not vary with the configuration of the block copolymers. The internal energy of the system primarily originates from the internal energy of the block copolymers, and the internal energy of the grafted homopolymer brushes is minuscule compared to that of the block copolymers. This suggests that the internal energy of the system is greatly influenced by the configuration of the block copolymers. As the various architectures of AB, A_1_A_2_B, and A_2_B copolymers change, both the internal energy of the system and the internal energy of the block copolymer increase. When *f_a1_* = 0.2, both the internal energy of the system and the internal energy of the block copolymer diminish slightly. This is because a regular hexagonal phase emerges in the system. Meanwhile, the interfacial energy decreases. Among the various components of the free energy of the system, the interface energy is the most significant.

In order to further clarify the effect of entropy effect on the system, the entropy of polymer brushes and the entropy of block copolymer were calculated, respectively. They can be obtained by the following formula:

(23) $$\frac{S_{br}}{K_{B}}=\frac{1}{\bar{\varphi_{h}}}[\bar{\varphi_{h}}\ln(\frac{Q_{h}}{V\bar{\varphi_{h}}})+\frac{1}{V}\int W_{a}\varphi_{a}dr]$$

(24) $$\frac{S_{co}}{K_{B}}=\frac{1}{\bar{\varphi_{co}}}[\bar{\varphi_{co}}\ln(\frac{Q_{co}}{V\bar{\varphi_{co}}})+\frac{1}{V}\int \left(W_{a}\varphi_{a}+W_{b}\varphi_{b})dr\right]$$

As Figure 6c reveals, the entropy of the block copolymer and polymer brushes remains inconspicuous as the value of *f_a1_* increases. This observation suggests that the entropy effect plays a minimal role in determining the ultimate structure of the system. When the volume fraction of polymer brushes remains constant, the block copolymers experience minimal distortion. Nevertheless, there is a minor increase in the entropy of both the polymer brushes and block copolymer. However, the entropy effects brought about by variations in polymer composition have minimal influence on the free energy, regardless of whether it is the layer structure or columnar structure. Nevertheless, in their absence, ordered structures are challenging to establish, as the entropy of polymer brushes is considerable. It turns out to be advantageous for achieving a stable and ordered system structure.

**Figure 7:**
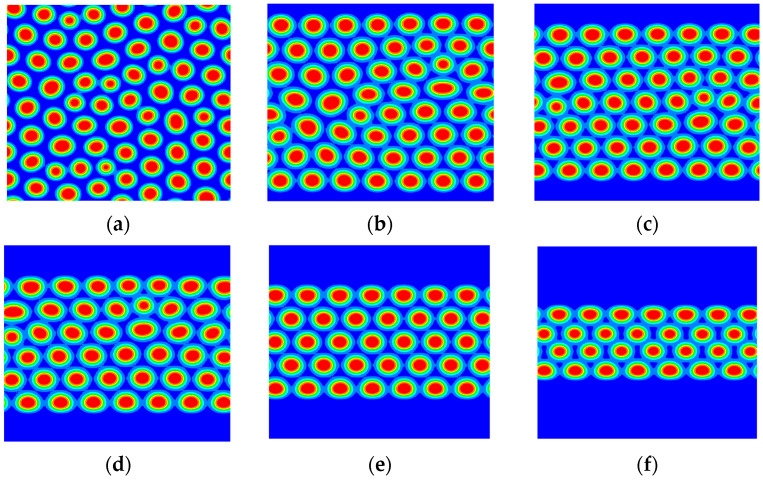

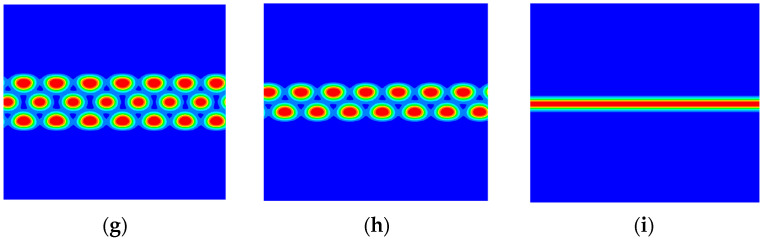
The morphology for the B phase of the A_2_B heteroarm star block copolymer with increasing the grafting density σ; (**a**) σ = 0.0, (**b**) σ = 0.15, (**c**) σ = 0.3, (**d**) σ = 0.4, (**e**) σ = 0.5, (**f**) σ = 0.6, (**g**) σ = 0.7, (**h**) σ = 0.8, (**i**) σ = 0.9. The blue and red areas denote A and B, respectively.

Furthermore, the pattern of the A_2_B heteroarm star copolymer was investigated by increasing the density of the polymer brushes. Figure 7 displays the morphology for the B phase of the A_2_B heteroarm star copolymer with an increase in the grafting density σ. The domain morphology changes from core-shell hexagonal phases to lamellar phases, and the layer decreases step by step. When the grafting density σ is 0.6, the system forms the four-layer hexagonal phase (as shown in Figure 7f). The four-layer hexagonal phase will change from three-layer to two-layer with increasing the grafting density σ. When the grafting density is large enough, the lamellar phase emerges. Indeed, increasing the grafting density leads to an increase in the brush height, thus a smaller gap for the block copolymers.

**Figure 8:**
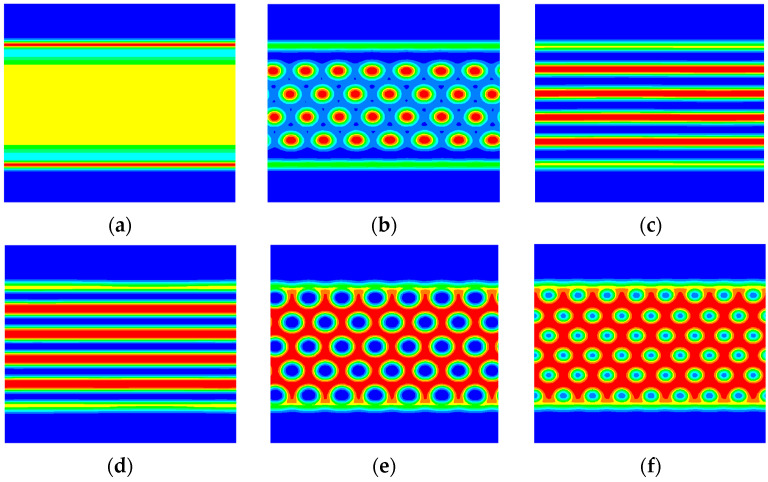
The morphology of block copolymers increases the volume of fraction A when the volume fraction of the polymer brushes is 0.4. The red and blue area denotes the fractions A and B, respectively. (**a**) $f_{a}$ = 0.2, (**b**) $f_{a}\;$= 0.3, (**c**) $f_{a}$ = 0.4, (**d**) $f_{a}\;$= 0.5, (**e**) $f_{a}$ = 0.6, (**f**) $f_{a}$ = 0.7.

If the other restricted conditions are constant, then the equilibrium phase topography of the system is determined by the block ratio. Figure 8 shows the morphology of A_2_B heteroarm star block copolymers with increasing the volume of fraction A when the volume fraction of the polymer brushes is 0.4. In Figure 8a, the layered phase separation in the system is not very obvious when the volume fraction of the A block copolymer is 0.2. The block copolymer forms the layered structure under the strong infiltration of the polymer brushes. When the volume fraction of the A block copolymer is 0.3, the restricted system in a suitable columnar arrangement occurs, as shown in Figure 8b. The domain-scale size of each column is essentially equal. In Figure 8c,d, the concentrations of A components and B components are almost the same, it is easy to form a layered structure when the volume fraction of the A block copolymer is 0.4~0.5. The concentrations of A components are less than the concentrations of B components to compared Figure 8c with Figure 8d, so the layered phase formed is slightly thinner. In Figure 8e,f, as the concentrations of A components are further increased to 0.6 or 0.7, the system forms a hexagonal column structure due to the influence of the interface energy.

The asymmetry observed in Figure 8b compared to Figure 8f in the B phase domains is attributed to the selective infiltration of the block copolymer influenced by the polymer brushes. Even without complete phase separation in the system, it still forms a layered structure close to the polymer brush surface to minimize surface energy when $f_{a}\;$= 0.2. This phenomenon breaks the symmetry due to the infiltrating effect of the polymer brushes. Therefore, it is not necessary to form a layered structure of the polymer brushes. To ensure adequate contact between the block copolymer and the polymer brushes, the interfacial phase of the polymer brushes and the block copolymers form a wavy surface with the polymer brushes’ soft nature. This reduces the free energy of the system. The formation of a layered structure often benefits the conformational entropy of the polymer brushes and the surface energy of the components and polymer brushes. However, it is not beneficial for the interface energy of the asymmetric block copolymer A2B. Consequently, under the competition of these three energies, the system transitions from hexagonal columnar to layered to hexagonal columnar phase.

### 3.2. The Effect of Colloidal Nanoparticle Dispersion on Phase Separation of Copolymers

The phase behavior of the system of A_2_B block copolymers and colloidal nanoparticles was investigated within two plates with grafted homopolymer brushes. In this section, the case of colloidal nanoparticles of $r_{p}=0.3R_{0}\;$ was considered. Previous studies have shown that large nanoparticles tend to be distributed in the middle of the polymer domain, while smaller nanoparticles tend to be distributed at the interface of the polymer. First, the case was considered in which the concentration of the colloidal nanoparticles and the grafting density of the polymer brushes were invariant. If the copolymer is confined in the parallel plates which were with charged colloidal nanoparticles suspension, it will make the polymer form a different layered crystalline state between two plates with colloidal nanoparticles.

**Figure 9:**
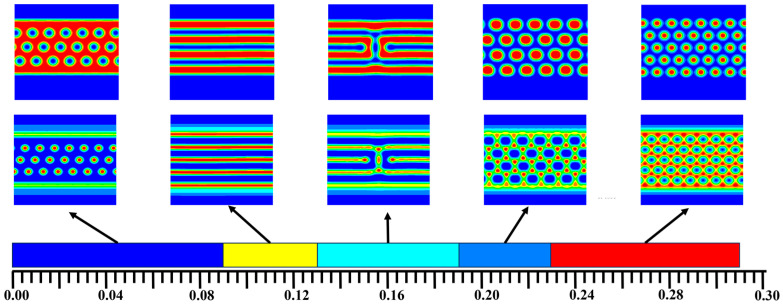
The concentration of nanoparticles $\phi_{p}$ = 0.15, the volume fraction of polymer brushes $\phi_{h}$ = 0.4, the system and the colloidal nanoparticles phase diagrams change with increasing the volume of fraction A. The blue and red areas denote the A and B in the first line, respectively. The red area denotes the nanoparticles in the second line. The concentration of colloidal nanoparticles $\phi_{p}$ = 0.15, the volume fraction of polymer brushes $\phi_{h}$ = 0.4. Blue bar: *f_a1_* ≤ 0.09, yellow bar: 0.09 < *f_a1_* ≤ 0.15, cyan bar: 0.15 < *f_a1_* ≤ 0.19, light blue bar: 0.19 < *f_a1_* ≤ 0.23 and red bar: 0.23 < *f_a1_* ≤ 0.29. The nanoparticles are in affinity with the A phase.

Figure 9 shows the system and the colloidal nanoparticles phase diagrams change with increasing the volume of fraction A. Here, the concentration of particles $\phi_{p}$= 0.15, and the volume fraction of polymer brushes $\phi_{h}$= 0.4. When the concentration of polymer brushes is 40% and the concentration of colloidal nanoparticles is 15% in the system, the concentration of the block copolymer is 45%. In Figure 9, the B phase was a cylinder structure dispersed in the A phase with the volume of fraction A increasing to 0.18. With the volume of fraction A increased to 0.3, the A_2_B block copolymers self-assembled into a layered structure. When the volume of fraction A increased to 0.38, the layered structure is disturbed gradually. Increased the volume of fraction A to 0.46, the B segment presents hexagonal column distribution whereas with large nanoparticles. Increasing the volume of fraction A to 0.6, the copolymer which is the star copolymer forms the hexagonal structure.

**Figure 10:**
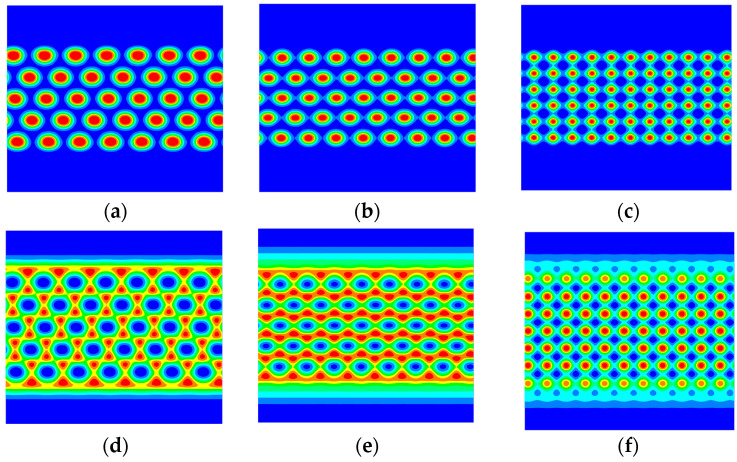
The B segments phase morphology with increasing the concentration of colloidal nanoparticles $\phi_{p}$ in the fixed block copolymer $f_{b}\;$= 0.4. Figure (**a**–**c**) showed the B segments phase morphology with increasing the concentration of colloidal particles $\phi_{p}$. The red area denotes the B phase. The blue area denotes the A phase. (**a**) $\phi_{p}$ = 0.1, (**b**) $\phi_{p}$ = 0.22, (**c**) $\phi_{p}$ = 0.29; (**d**–**f**) showed the colloidal nanoparticles phase topography with increasing the concentration of colloidal particles $\phi_{p}$. The red area denotes the nanoparticles clearly. (**d**) $\phi_{p}$ = 0.1, (**e**) $\phi_{p}$ = 0.22, (**f**) $\phi_{p}$ = 0.29.

In Figure 10, when *f_a1_* ≤ 0.09, colloidal nanoparticles tend to be distributed in the middle of the domains of A segment when A block is a hexagonal distribution. When 0.09 < *f_a1_* ≤ 0.15, the block copolymers form the layered distribution, and the nanoparticles tend to be distributed in the A phase, and colloidal nanoparticles also form a layered structure. When 0.15 < *f_a1_* ≤ 0.19, the layered structure was disturbed, and the nanoparticles tended to be distributed in A phase and B phase. When 0.19 < *f_a1_* ≤ 0.23, the block copolymers formed a hexagonal arrangement, the nanoparticles surrounded by A segment and tend to be distributed in B phase. When 0.23 < *f_a1_* ≤ 0.29. the block copolymers also formed a hexagonal arrangement, and the colloidal nanoparticles filled the gap of the B segment which was mainly around the B segment. Interestingly, the grafted chain may lead to long-range repulsions between the particles, which can stably order the nanoparticles and control the symmetry of the structure in the desired direction. Due to the limitation of polymer brushes, two block copolymers will produce from layer to column to layered transition. The addition of particles is conducive to the formation of orderly layered or columnar phases, which is conducive to phase separation. This has a certain guiding significance for nanometer-etching technology.

Previous studies on the mixture system of block copolymers/colloidal particles showed that, when the block ratio of the diblock copolymers changes, the distribution of colloidal particles in the system is different. Finally, the case was considered in which the concentration of the colloidal nanoparticles and the grafting density of the polymer brushes are invariant. Figure 10a–c showed the B segment phase morphology with increasing concentrations of colloidal nanoparticles $\phi_{p}$; Figure 10d–f showed the colloidal nanoparticles phase topography with increasing the concentration of colloidal particles $\phi_{p}$. The volume fraction of fraction B is $\;f_{b}$= 0.4.

The change in the volume fraction of colloidal particles is also responsible for altering the phase behavior of the system. In Figure 10a,d, when the volume fraction of the colloidal nanoparticles is $\phi_{p}$ = 0.1, they form a hexagonal phase and are uniformly distributed within the A phase. As the volume fraction increases to $\phi_{p}$ = 0.22 in Figure 10b,e, the nanoparticles continue to arrange in a hexagonal pattern and are still evenly distributed within the A phase. However, when the volume fraction reaches $\phi_{p}$ = 0.29 in Figure 10c,f, a significant transformation occurs. The phase of the colloidal nanoparticle dispersion diminishes, and interestingly, the nanoparticles are now dispersed in the B phase. The morphology of the system’s phase is irregular, and the nanoparticles exhibit a novel square arrangement. The addition of colloidal nanoparticles has a profound impact on the phase behavior of the system. As the polymer brushes infiltrate the nanoparticles, they congregate in regions close to the polymer brushes, with those in the central area arranged hexagonally around component B. As we know, for nanoscale colloidal particles, limited suspension is a common route to forming colloidal crystals. For instance, when a colloidal polymer mixture is confined between two parallel hard plates, a layered structure may emerge; however, the particles’ surface structures tend to be evenly distributed or densely packed.

## 4. Discussion

In summary, the impact of colloidal nanoparticles on the phase transitions of amphiphilic copolymers, such as the linear diblock A_1_A_2_B and the heteroarm star copolymer A_2_B, confined between two polymer brush substrates was studied using a real-space self-consistent field theory. As the fraction of block A_1_ increases, a series of structural transformations occur, progressing from lamellar to core-shell hexagonal phases. These transformations are primarily influenced by the wetting properties of the grafted polymer brush surface and the architecture of the copolymers. The film thickness of the AB block copolymer conforms to a three-layer lamellar structure, while the A_2_B heteroarm star copolymer film assumes a four-layered cylinder phase. By increasing the density of the polymer brushes, the pattern of the A_2_B heteroarm star block copolymer transforms from core-shell hexagonal phases to lamellar phases, with a corresponding decrease in layer thickness. The results indicate that the star architecture serves as a strong topological constraint that regulates the geometry of the microphases. The block sequence plays a crucial role in microphase formation. The entropy effect caused by changes in polymer architecture has minimal impact on the free energy. The formation of the core-shell hexagonal phase is attributed to adjustments in the grafting interface shape, which is determined by the characteristics of the polymer brushes.

The impact of nanoparticles on the system was thoroughly examined, with a specific focus on nanoparticle dispersion. It was observed that the introduction of particles promotes the formation of organized layered or columnar phases, thereby facilitating phase separation. The findings indicate that the phase behavior of the system is strongly influenced by the polymer architecture and the number of colloidal nanoparticles. The combination of colloidal nanoparticles and the soft confined surface of polymer brushes makes it easier for amphiphilic AB copolymers to form ordered structures. The soft surfaces of polymer-grafted brushes, along with the conformation of the block copolymers, work together to ensure even dispersion of the nanoparticles. Experimental results support this conclusion [52,53,54]. These findings provide valuable guidance for the development of novel functional materials using nanoetching technology, nano-photoresist, and nanoprinting.

## Data Availability

Data are contained within the article.
